# Supplementary material for: Fragility Index, power, strength and robustness of findings in sports medicine and arthroscopic surgery: a secondary analysis of data from a study on use of the Fragility Index in sports surgery
Source: PeerJ. 2019 May 24;7:e6813. doi: 10.7717/peerj.6813 (PMC6536113; doi:10.7717/peerj.6813)
Supplement: Supplemental Information 1 [file peerj-07-6813-s001.docx]

**Two studies with data inconsistency**

Study by Rafols et al was excluded from the analyses. Studies by MacDonald et al. and Lund et al. had reporting inconsistencies.

*Rafols et al.*

Appendix 2 in the original study by Khan et al states that the study by Rafols et al has a sample size of 67 patients with 40 total events. The only dichotomous variable with a significant finding mentioned in the abstract is the presence of effusion in the MRI at the 6 months follow-up.

First paragraph in the results states that 67 patients were eligible for the study; 10 were not included resulting to 30 patients in the group I and 27 patients in the group II. The last paragraph in the results states “*In total, 36.7% of patients in group I did not present with effusion compared with 21.1% of patients in group II. This difference was significant (P < .05) (Fig 3)*”. Rafols et al. do not report the corresponding number from the which result is calculated.

36.7% of the 30 patients in the group I equals 11 patients (exact 11.01 patients). 21.1% of 27 patients in the group II equals to 6 patients (exact 5.697). If we look at the patients with effusion, we have 30 – 11 = 19 patients in the group I and 27 – 6 = 21 patients in the group II. This totals to 40 patients, the same number reported in the appendix of the original study.

|  | Group I | Group II |
| --- | --- | --- |
| No effusion in the MRI | 11 | 6 |
| Effusion in the MRI | 19 | 21 |

From this 2 x 2 contingency table neither the Fisher exact test or chi-square test produce a significant finding (p=0.2621, p=0.368, respectively).

*Lund et al.*

Appendix 2 in the original study by Khan et al states that the study by Lund et al has a sample size of 51 patients with 17 total events. The abstract includes two dichotomous variables with a significant finding: a positive pivot shift test and a presence of anterior kneeling pain.

Third paragraph in the methods states that 51 patients were randomized resulting to 25 patients in the BPTP group and 26 in the QTP group.

First paragraph in the results states “*A positive pivot shift test result at 1-year follow-up was seen significantly less in the QTB group than in the BPTB group - 14% and 38%, respectively (P= .03).*”. Authors state that a total 12 patients were lost but based on the figure 1 no patients were lost until the 1-year follow-up. 1 patient in the QTB group was excluded due to revision surgery.

14% in 26 patients or 38% in 25 patients equals to 3.64 or 9.5 patients. Rounding 9.5 to 9 will result to nominal significance.

|  | QTP group | BPTP group |
| --- | --- | --- |
| Positive pivot shift | 4 | 9 |
| Negative pivot shift | 22 | 16 |

Chi-square test, p=0.09

|  | QTP group | BPTP group |
| --- | --- | --- |
| Positive pivot shift | 4 | 10 |
| Negative pivot shift | 22 | 15 |

Chi-square test, p=0.048

Second paragraph in the results states: “*There was a significant difference in knee walking ability test results between the 2 groups, with 34% of the patients who received BPTB grafts in the 2 lowest scoring groups (impossible or difficult) compared with 5% in the QTB group (P=.03).*”.

The time point of this assessment is not clearly stated. 5% in 26 patients or 34% in 25 patients (1 year follow-up) equals to 1.3 or 8.5 patients, respectively. Depending on the rounding of 8.5, the p-value for Chi-square test is either 0.008 or 0.003. Since 9 patients is the “*best case scenario*” this was selected for the study analysis.

5% in 20 patients or 34% in 18 patients equals to 1 (exact 1.0) and 6 (exact 6.12) patients respectively. The chi-square test is significant at 0.05 level (p=0.02).
