# Supplementary material for: Fragility Index, power, strength and robustness of findings in sports medicine and arthroscopic surgery: a secondary analysis of data from a study on use of the Fragility Index in sports surgery
Source: PeerJ. 2019 May 24;7:e6813. doi: 10.7717/peerj.6813 (PMC6536113; doi:10.7717/peerj.6813)
Supplement: Supplemental Information 3 [file peerj-07-6813-s003.docx]

**Correspondence concerning studies with inconsistencies**

*Rafols et al*.: Multiple issues with data and statistical methods

Authors contacted July 13^th^, Aug 13^th^ and Sep 20^th^ 2018

*Lund et al*.: Data inconsistency

Authors contacted July 3^rd^, 25^th^ and Aug 20^th^ 2018

*Franceshi et al*.: Use of Wilcoxon signed-rank test to compare proportions

Authors contacted Aug 10^th^ 2018 and responded Aug 11^th^

*Armellin et al*.: Use of Fisher exact test resulting to a p-value >0.05

Authors contacted Aug 20^th^ and Sep 20^th^ 2018

*Lee et al*. Use of Fisher exact test resulting to a p-value >0.05

Authors contacted Aug 20^th^ and Sep 24^th^ 2018

*MacDonald et al*. Use of Fisher exact test resulting to a p-value >0.05

Authors contacted July 4^th^ 2018 and responded July 9^th^ 2018

*Zhang et al*. Use of Fisher exact test and Chi-square with or without Yates corrections resulting to a p-value >0.05

Authors contacted Aug 11^th^ 2018 and Aug 30^th^ 2018

*Zhang et al*. Use of Fisher exact test and Chi-square with or without Yates corrections resulting to a p-value >0.05

Authors contacted Aug 21^th^ 2018
